# Products of Conception: Imaging and Imagining Maternal–Fetal Relationships

**DOI:** 10.1007/s10912-025-09940-x

**Published:** 2025-03-26

**Authors:** Sabina Dosani

**Affiliations:** Medical and Health Humanities, University of East Anglia, Norwich Research Park, Norwich, NR4 7TJ UK

**Keywords:** Miscarriage, Ultrasound, Maternal attachment, Ghost, Obstetric, Scan

## Abstract

When my pregnancies ended in silence in an ultrasound suite, I was left with many questions that my medical training did not help me to answer. To investigate what an ultrasonically imaged embryo might represent in obstetric and maternal contexts, I turned to novels and memoirs, where I discovered that new traditions of writing about miscarriage and ultrasound are being crafted. In this paper, I consider the ghostly motifs in depictions of obstetric ultrasound in three contemporary works: *Queenie* (2019) by Candice Carty-Williams; Hilary Mantel’s memoir, *Giving up the Ghost* (2013); and Maggie O’Farrell’s personal essay “Baby and Bloodstream,” from *I am, I am, I am: Seventeen Brushes with Death* (2017). In each text, the ultrasound is a contested site where obstetric and maternal miscarriage narratives collide.

A pregnant woman has an ultrasound scan. COVID-19 restrictions in England in 2021 dictate that the woman attends alone. In the scanning room, she takes out her phone to film the ultrasound for her partner. “That’s illegal,” says the technician, pointing at a notice on the wall: “women must not record or take photographs during the ultrasound.”

Throughout the pandemic, pregnant women were prevented from taking images of their fetuses. A barrister from Doughty Street Chambers called the ban “unlawful.” The Society and College of Radiographers defended the ban, saying, “a pregnant woman holding a phone leads to a taut abdomen which makes scanning very difficult”.

In this argument between healthcare professionals and pregnant women, medical and legal narratives focus on ultrasound image ownership. Underpinning this is a complex story about what the ultrasound image represents and to whom: is it a clinical investigation or the first photograph of an anticipated baby?

My curiosity about this began during one of my own pregnancies. Between the births of my two daughters, I experienced recurrent miscarriages. At that time, I worked as a consultant psychiatrist, commissioned to conduct expert witness assessments of children subject to care proceedings. Almost all my professional conversations were about maternal attachment, about what makes a good-enough mother and what causes harm. In my personal life, I was alternately preoccupied with any hint of possible pregnancy or with signs that might herald impending fetal death.

Following those recurrent miscarriages, I became pregnant again, and, while under the care of the recurrent miscarriage clinic, I underwent fortnightly ultrasounds for the first 20 weeks of pregnancy. When I gave birth, I had a sense of “knowing” my daughter, after already “seeing” her so frequently.

At the recurrent miscarriage clinic, I increasingly noticed the oddities of medical language. For example, my obstetrician used military metaphors to describe his treatment plans. My immune system, he postulated, was “mounting an attack on embryos.” He suggested I had high numbers of “natural killer cells.” Throughout that pregnancy, I lived with my husband, an army officer, at a large military headquarters in London. War language was part of my life, but I had not expected it to be part of my pregnancy.

In an attempt to make sense of my experiences, I read widely. I found that in contemporary British obstetric literature, miscarriage is almost always presented as a biological process, akin to a disease state to be medically or surgically managed. The language I reached for as a medically qualified patient, especially relational frames of maternity and the language of grief, was at odds with the contemporary obstetric terminology of miscarriage, embryo, and fetus. I wondered, therefore, how other women write about similar experiences. I began to pay close attention to how women were describing obstetric ultrasound.

To examine women’s diverse experiences, I read contemporary texts from different literary genres, by women from different ethnic backgrounds, across the reproductive lifespan. I found that a new tradition of writing about ultrasound and miscarriage is being crafted in memoir, and also in fiction. For this paper, I selected three texts to present in detail: *Queenie* (2019) by Candice Carty-Williams; Hilary Mantel’s memoir, *Giving up the Ghost* (2013); and Maggie O’Farrell’s essay “Baby and Bloodstream,” from her autobiographical collection, *I am, I am, I am: Seventeen Brushes with Death* (2019). Double Booker Prize winner Hilary Mantel was in her sixties when she wrote her story of a non-traditional childhood in a conservative Catholic community, followed by formative years marked by misdiagnosis of endometriosis, with secondary infertility and concomitant psychological injuries. Among the ghosts Mantel writes about are those of her unborn children.

O’Farrell was in her forties when *I am, I am, I am*, in which she writes about the ultrasound scan that confirmed her missed miscarriage, was published. In the chapter “Baby and Bloodstream,” O’Farrell also writes about “the ghost children we still carry in our minds, the ones who didn’t make it” (O’Farrell 2019, 105). The intertextuality between O’Farrell’s essay and Mantel’s memoir represents a conversation between women sharing a grief that society is not yet good at recognizing.

Carty-Williams was in her twenties when she wrote *Queenie*. Published at the height of the #MeToo campaign and at the beginning of the Black Lives Matter movement, *Queenie* would be genre-defining even if it did not begin with a missed miscarriage.^1^ I chose *Queenie* because Carty-Williams tells heartbreaking stories with humor and specifically because she uses this humor to challenge the obstetric discourse. *Queenie* is presented as fiction, and Mantel’s narrative bears the subtitle, “a memoir.” Yet, both books transgress traditional boundaries between auto-fiction, memoir, and the traditional novel.

All three literary works share an intimacy of voice and stylistic directness that contrasted with the obstetric discourse. All three were critically and commercially successful, suggesting that the language used to depict fetal-maternal relationships resonated not just with me, but with literary judges and also with many readers. O’Farrell, Carty-Williams, and Mantel all challenge assumptions about what fetal loss looks and feels like from a woman’s point of view in obstetric clinical spaces.

In my textual-critical enquiry, I noticed a pattern: life-altering revelations occur when each protagonist sees into her uterus in an ultrasound suite. Both O’Farrell and Carty-Williams open with scenes in which the protagonist finds out unexpectedly during an ultrasound scan that she is carrying a deceased embryo. In Mantel’s chapter “Show Your Workings,” describing her endometriosis and subsequent loss of fertility, she describes becoming aware of the latter during an ultrasound examination.Within the texts, I found a new cluster of motifs about the ghostly presence of ultrasonics.

Carty-Williams opens her story with Queenie texting her boyfriend: “In the stirrups now. Wish you were here,” as she undergoes a transvaginal ultrasound during a routine check of her intrauterine contraceptive (2019, 1). This sex joke represents the transformative moment when Queenie’s body changes from a libidinal body to a maternal body. Moments later, her maternal body becomes the site of a death:

‘Okay,’ she uttered, after a pause and a prod. ‘I’ve asked another doctor for a second opinion. And having had another look, it’s just that - well, is there any chance you were pregnant, Queenie?’

I sat up again; my stomach muscles would be shocked into thinking that I was exercising at this rate.

‘I’m sorry, what do you mean?’

‘Well,’ the doctor said, peering at the ultrasound, ‘it looks like you’ve had a miscarriage.’ (Carty-Williams 2019, 7)

From a medical perspective, the scan evidences the scientific triumph of the intrauterine device:

‘It can happen with most forms of contraception,’ she told me clinically, her eyes that I’d previously thought were kind still fixed on the screen. ‘Most women don’t know about it. At least it’s done the job.’ (Carty-Williams 2019, 13)

This clinically accurate pronouncement smothers any possibility for Queenie to express her emotions. The doctor’s blunt statement is immediately followed by Queenie lying on a clinical bed, remembering how her ex-partner’s grandmother speculated about future children. It reads as a quasi-natal scene, “‘Oh you two will have beautiful children,’ Tom’s grandmother said, staring at us from across the table. Joyce had cataracts, but she could still see the future, it seemed” (Carty-Williams 2019, 13).

Queenie’s word “children” is a lexical choice suggesting that the scanned image represents the loss of a relational other. This is discordant with her clinician’s terminology: “Apparently all of the fetal tissue has gone, lovely” (Carty-Williams 2019, 61). These divergent medical and maternal reactions represent different interpretations of ultrasonic confirmation of embryonic demise. Imaging and imagining are two distinct poles on the axis of understanding what is happening in the expectant woman’s uterus.

Queenie’s fantasies deliver her future imagined babies and the imaged deceased embryo into the clinic. After the scan, in the familiarity of her home, Queenie imagines her embodied pregnancy: “‘Would I have been ready,’ I asked myself aloud, stroking my stomach. My mum was twenty-five when she got pregnant with me” (Carty-Williams 2019, 13). The juxtaposition of the doctor’s pronouncement and Queenie’s fantasy makes sense. Of course, Queenie is thinking about babies. She has just been an eyewitness to her embryo’s existence.

The ultrasound screen is both the projection site of the image of Queenie’s embryo and an exterior, observing eye, extending the medical gaze. For example, Queenie, lying on the examining couch, is “desperate for her [the doctor] to look at me, to acknowledge that this news might have affected me in some way.” But the doctor does not look at Queenie when she breaks the news about the miscarriage: instead, “her eyes that I’d previously thought were kind were still fixed on the screen.” When Queenie’s doctors focus on the images, they stop seeing her as a person and can barely remember her name: “‘So what do you do…Queenie?’ the doctor asked, glancing at my chart” (Carty-Williams 2019, 7).

This erasure can be fatal. Queenie’s encounter is a testimony to the dangerous reality of Black women in obstetric spaces. Black British women are almost five times more likely to die from pregnancy-related causes.^2^ Throughout the novel, Carty-Williams uses humor to challenge aspects of medical culture that make these tragic deaths more likely. For example, in this ultrasound examination scene, Carty-Williams employs techniques from absurdist observational comedy to draw attention to the obliterating effect of the medical discourse on Queenie:

‘Let’s get a closer look…’ Dr Smith said, bending down and peering between my legs. ‘What’s wrong? Can you not find it?’ I asked, worried that the coil had maybe absorbed into my womb, the way that I still worried that every tampon ever inserted was still knocking about inside me.

‘What do you think, Ray?’ the first doctor asked her colleague. ‘We might need to get Dr Ellison in here, you know,’ Dr Smith replied, standing back up and putting his hands on his hips.

‘I saw a cleaner mopping up some sick in the hallway, why don’t you get him in here to have a look as well?’ I asked all three hospital staff as they stared at the ultrasound image. (Carty-Williams 2019, 7)

This exchange highlights the discordance of being intimately probed, while Queenie’s questions hang in the air, unanswered. Queenie becomes a womb-on-legs, addressed to reposition herself for clinical convenience, directed to “hop down” and “hop back up” (Carty-Williams 2019, 3). As soon as the doctor has made her detached remarks, Queenie laments her loss of choice:

I wasn’t going to have a baby. Obviously. But it would have been nice to have had the choice. Having a contraceptive placed in my body wholly suggests that I was not wanting to have a baby, so yes, my choice would be to not actually carry a child to term and then raise it, but that isn’t the point. (Carty-Williams 2019, 13)

Queenie cannot unsee her maternal body. For example, when relating a casual sexual encounter to her friend, Queenie refers to “babies”: “‘We were careful,’ I lied. I opened my mouth to speak again, knowing that I should probably tell her about the miscarriage, ‘No sleazy babies on the way’” (Carty-Williams 2019, 59). Queenie’s language is evocative of her uncertainty, arising from tensions between the gynecological account (contraceptive success) and a maternal one (what would my babies look like?).

In O’Farrell’s biographical essay, an even more explicit oscillation of perspectives between the clinical and maternal, and the resultant tension, is apparent from the opening lines of “Baby and Bloodstream” in the middle of an ultrasound examination in which the narrator receives news of a missed miscarriage. She describes the scanned image in terms that are undeniably human: “I look again at the image of the baby on the screen. There it is. Sitting up in its dark cave, as if waiting for something, as if on its best behavior. If I sit straight, it seems to be saying, no one will notice” (O’Farrell 2019, 95).

O’Farrell’s description of her baby “as if” waiting in the dark alludes to lost maternal possibilities. The ultrasound image creates both the illusion of the child and O’Farrell’s maternal imagination. O’Farrell’s descriptions ascribe to the fetus not just a corporeal self, but one that takes on the form of a developmentally older infant, capable of sitting up. By imagining this advanced developmental state, O’Farrell suggests mothering this specific child in the future. O’Farrell’s narrative style when imagining this impossible possibility is marked by an abundance of physical detail, reminiscent of a mother describing a living child: “It will be blond, it will be dark, auburn, curly haired. It will be tall, it will be petite. It will look like you, its father, its brother, a mélange of all three” (O’Farrell 2019, 99).

After seeing her scanned womb, the baby exists not just in O’Farrell’s fantasies of a child with auburn or curly hair, but as a distinct person, with relational possibilities. Her first mention of her unborn, “in its dark cave, as if waiting for something,” suggests that one of those relational possibilities is mourning the fetus in her womb-tomb. I read this also as an allusion to how O’Farrell was “in the dark,” assuming herself to be carrying a healthy baby, until the darker truth was ultrasonically visible.

After imagining the physicality of her conceptus, O’Farrell references an embodied maternal relationship, for example: “You will take it swimming, you will rake leaves and light bonfires, you will push it along the seafront, you will tuck it into the basket its brother used” (O’Farrell 2019, 99).

Ongoing pregnancy symptoms make it difficult for O’Farrell to come to terms with the fetal demise:

You will walk around looking pregnant, feeling pregnant, to all intents and purposes still pregnant, but the baby is dead. Sometimes your physiological inability to process the death of the fetus infuriates you, devastates you, at others it seems only apt, sane. Why give up, your body is saying, why let go, why accept this end? (O’Farrell 2019, 101)

O’Farrell’s title, “Baby and Bloodstream,” references the bloodstream by which her body will expel the fetus. It also refers to the maternal bloodstream into the placenta. Pairing the baby and bloodstream not only alludes to that essential physical bond but also raises possibilities of death. Writing about the emotional aftermath of her ultrasonically diagnosed miscarriage, she says, “Your body has failed at this most natural of functions; you can’t even keep a fetus alive; you are useless, you are deficient as a mother, before you were even a mother” (O’Farrell 2019, 100). Her repetition of the word *mother* implies maternal failings, highlighting the relational conundrum of mothering a fetus before attaining the social status of motherhood.

Neither Queenie’s doctor nor O’Farrell can take their eyes off the ultrasound screen, but for different reasons. Queenie’s doctor is conducting routine imaging; she does not need to glance up, yet for O’Farrell, the scan is an introduction to her baby: “I can’t look away from the screen, even when the radiologist starts talking again, even when they say I can get dressed. I want to burn that image of that tiny ghost-pale form into my retina” (O’Farrell 2019, 95).

O’Farrell cannot look away because the screen carries the projection of the enormity of her loss, writing, “It seems to me that pregnancy at any stage is significant, life-changing enough to warrant telling those closest to you…How else do you explain the grief, the stunned pain on your face, the tears, the shock” (O’Farrell 2019, 98). While their respective clinicians’ images “retained products of conception,” O’Farrell and Queenie imagine their unborn, never-to-be-born, children. O’Farrell depicts the emotional meaning for the woman undergoing the scan: “You must adjust to this new picture. You must give it all up” (O’Farrell 2019, 103).

In both O’Farrell’s adjustment to maternal loss and Queenie’s expressions of uncertainty, the ultrasound is a significant stimulus. Queenie imagines the physical appearance of the child she is not having, by recalling her partner’s grandmother, Joyce’s racially charged language: “Your lovely soft brown skin, Queenie, but lighter. Like a lovely milky coffee. Not too dark!” (Carty-Williams 2019, 7). Similarly focusing on imagined physical details, O’Farrell writes, “Gone is the child with the blond or dark or auburn hair; gone is the person they might have been, the children they themselves might have had” (O’Farrell 2019, 100).

O’Farrell prefaces her essay “Baby and Bloodstream” with sketches from *The Midwives Book* (1671), written by the midwife Jane Sharp (O’Farrell 2019, 93). The Wellcome Library in London has records of 82 midwifery manuals published between 1540 and 1699. Eighty-one are written by university-educated men with limited experience of attending laboring women. Sharp wrote in English, rather than Latin or Greek. Her drawings and humor made her manual accessible to midwives lacking formal education and to pregnant women.

In presenting Sharp’s 450-year-old images, O’Farrell frames her essay as part of the long-established tradition of women writing about female bodies, contributing to democratized knowledge. Sharp’s images remind us of the tensions that have been part of reproductive cultures for centuries: Who possesses knowledge about the unborn in the womb? Who holds the power to make and distribute such images?

Common medical terms like “missed miscarriage” or “retained products of conception” afford no space for maternal grief. O’Farrell’s conflation of ultrasonic image and ghost reads as an act of memorialization: “I want to burn the image of that tiny, ghost-pale form into my retina. I want to remember it. To honor its existence, however short” (O’Farrell 2019, 95).

Ultrasonically rendered images, like ghosts, depend on extra-sensory perception: technological for an ultrasound image and metaphysical for the ghost. What O’Farrell could not see, she imagined, transporting her unborn’s portrait from ultrasound image to ghost:

To dismiss a miscarriage as nothing, as something you take on the chin and carry on, is to do a disservice to ourselves, to our living children…to those ghost children we carry in our minds, the ones who didn’t make it. (O’Farrell 2019, 105)

O’Farrell’s word “carry” is unmistakably maternal. Physically supporting a fetus and then providing bodily and psychological containment to a child are what a mother does. O’Farrell’s “carrying” could be considered the opposite of Mantel’s “giving up”; however, I read Mantel’s “giving up” as maternal as well, albeit an act of thwarted maternity. Unmarried women of Mantel’s generation, and in particular those sharing her strict Irish Catholic heritage, spoke about “giving up” babies for adoption. Even today, the adoption organization Coram writes, “If you decide to give up your baby for adoption you will no longer have parental responsibility for them.”^3^

The connection between the two texts, O’Farrell’s text can be read as an inversion of Mantel’s, is further supported by a direct allusion. Immediately, after her paragraph on carrying, O’Farrell overtly references Mantel:

During the week in which a miscarried baby of mine would have been born, I found the following passage in Hilary Mantel’s memoir, *Giving Up the Ghost*:

[*Children’s*]* lives start long before birth, long before conception, and if they fail to materialize at all, they become ghosts in our lives…The unborn, whether they are named or not, whether or not they are acknowledged, have a way of insisting: a way of making their presence felt.* (O’Farrell 2019, 105, italics quoting Mantel mine)

Although both writers invoke ghost children as a proxy for memorialisation, Mantel uses the ghost story as a frame for her entire memoir, with supernatural beings haunting moments of otherwise calm domesticity. For example, Mantel opens her memoir by introducing readers to her dead stepfather, Jack: “I know it is my stepfather’s ghost coming down. Or, to put it in a way acceptable to most people, I ‘know’ it is my stepfather’s ghost” (Mantel 2013, 1).

Reading this description that is so far from ordinary, I am reminded of what O’Farrell writes about her missed miscarriage: “It is not ordinary to conceive a life and to lose it. These passings should be marked, should be respected, should be given their due. It is a life, however small, however germinal” (O’Farrell 2019, 104). O’Farrell’s out-of-the-ordinary loss references Mantel’s extraordinary experiences. There are echoes of Mantel’s ghost child too, in O’Farrell’s description: “These people, spirits, wraiths who never breathed air, never saw light. So invisible, so evanescent are they that our language doesn’t even have a word for them” (O’Farrell 2019, 105). Her supernatural adjectives encapsulate the impossible task of describing miscarried fetuses, whose brief existence is confirmed and preserved by a shadowy image. Her use of the word “wraith,” defined in the Oxford English Dictionary as “a ghost or a ghostly image of someone, especially someone seen shortly before or after their death,” offers the possibility of memorialisation. O’Farrell’s ghost has stepped out of the ultrasound, fully formed, precisely because the child, as imagined by O’Farrell, predates any physical conception.

Reading these texts, I also became aware of a new, self-referential tradition within the memoir of writing about the unborn. The influence of Mantel’s writing on O’Farrell is apparent. Mantel’s epithet to her memoir comes from Judy Jordan’s poem “Sharecroppers Grave”: “My children who won’t hear/ The night full of cries they will never make.” This epithet suggests the ghosts of babies that Mantel imagined but can never bear. The night evokes the darkness of the ultrasound. Her children’s cries are beyond audible reach, like ultrasonic waves, yet leave ghostly traces. Mantel’s ghosts are visitors from an unlived past and also from an impossible future. In immortalizing her imagined children, she condemns herself to remaining haunted.

I have come to understand *Giving up the Ghost* as a folkloric expedition where the reader is taken on a journey through not only “what might have been” but also “who might have been.” If Mantel’s ghost children can be said to have a moment of conception in her memoir, it is the depiction of undergoing preoperative ultrasound at St George’s Hospital. Obstetric ultrasound was not commonplace when Mantel was an in-patient in 1979. Mantel recalls the journey across London, “I needed to have an ultrasonic scan. For this I needed to cross London. Saint George’s Hospital at Hyde Park Corner was in its last weeks of occupation…for the high-tech stuff I needed to go to the new St George’s at Tooting” (Mantel. 2013, 195).

I was surprised to find that the birthplace of Mantel’s ghost children was an ultrasound suite. Given that ultrasound was a first-trimester rite of passage when O’Farrell and Queenie were writing, it is understandable that both of those more recent texts foreground visions of mothering with scenes from the scanning suite. It did surprise me that Mantel employs a similar narrative sequence in her chapter, “Show Your Workings,” because she writes about events that occurred a decade before obstetric ultrasound had become a first-trimester rite of passage for British women. Nevertheless, Mantel foreshadows the loss of her fertility with a technological exposure: a junior doctor mistakes her distended abdomen for a sign of pregnancy and investigates his misconception using ultrasound:

The Senior Registrar examined me and thought I was pregnant. He winked at me. That’s a baby in there, he said, confidently patting my swollen abdomen. He ran off to get a fetal heart monitor. But there was no baby. Not Catriona, not Modestine: not anyone, only the ghost of my own heartbeat, amplified to the outside world. ‘Oh well,’ the registrar said. ‘Looks like I was wrong, eh.’ (Mantel 2013, 194)

Mantel then laments her lost maternity through a description of a second ultrasound:

He showed me the blossoming growths around my ovaries. For the first and last time, I saw my womb, with two black strokes, like skilled calligraphy, marking it out: a neat diacritical mark in a language I would never learn to speak. (Mantel 2013, 201)

Reading this aloud, I heard “killed calligraphy” and wondered if a murderous allusion is intentional. Diacritical marks are not usually used in English. Perhaps, Mantel is referencing the language of maternity, ever lost to her. After the ultrasound, Mantel’s fertility was surgically excised, but her maternal emotions were not dissected out. This made Mantel into a ghost mother, with her maternal impulses directed towards her (now impossible) children “whose lives begin long before they were born.”

If the ultrasound image has become a contemporary icon of life, it also serves as a symbol of fecundity. Mantel’s experience in the ultrasound suite haunts her because it is the moment of transfiguration for her (ghost) children, from potentiality to (im)possibility. Mantel may have imagined having children at an earlier time, suggested by the names she has already chosen, but the ultrasound conferred material proof of their non-existence, both at the time and in the future. Unlike O’Farrell, Mantel cannot write a redemption story, so she carries her ghost children.

For years, Mantel’s symptoms of endometriosis were dismissed by doctors as “all in the mind.” She confronted her general practitioner about her experiences of late diagnosis and sterilization. There is no plea to her reader for sympathy, just facts. “I went back to the GP who had been treating me, or failing to treat me, downtown.” There is no sympathy, either, from the GP, nor any regret for his negligence. With remarkable cruelty, he twists the knowledge of Mantel’s sterility, presenting it as advantageous to her, absolving himself of culpability. “Oh well,” he said. He shuffled his own sandalled feet under his desk. “There’s one good thing anyway. Now you won’t have to worry about birth prevention” (Mantel 2013, 211).

Ghost stories are often about unresolved disappearances. Mantel’s disappearing fertility remains unresolved because of her ongoing maternal emotions. Her phrase “there wasn’t much to be done, by the stage I’d reached,” calls to mind the expected stages of womanhood that would have been part of her convent education. When she arrived on the gynecology ward, Mantel was a young wife. The Catholic cultural expectation was that motherhood would follow. Mantel’s grief at finding that option taken from her haunts her during other life stages:

What I would have liked was a choice in life. Leisure, to reverse my earlier decision that children didn’t matter to me, to ask if the circumstances of my mind had changed. The time I fell in love is the time I should have acted, and now that era of my life is over, and my school-friends are becoming grandmothers, I miss the child I never had. I know what Catriona would have been like. (Mantel 2013, 227)

Keeping her ghost child alive, “seeing” her, and devoting attention to her may be an unconscious attempt on Mantel’s part to repair her regretted lack of maternal longing during her twenties. Mantel cannot go back in time to canvass doctors more urgently, receive an earlier diagnosis, and restore her fertility. Instead, a shadow of these events happens metaphysically.

Mantel’s ghosts offer a resolution to an impossible dilemma: how to maintain her maternal relationship with her children, conceived only in her mind. In my work as an expert witness, the ability of a mother to form a mental image of her child is an important aspect of healthy maternal attachment. Mantel’s ghost suggests enduring maternal bonds to the baby that could have been. The memoir provides her with the same seemingly impossible thing that the ultrasound image offers: a depiction immortalizing the baby as a non-perishable being.

These three texts enabled me to examine the obstetric gaze from the critical perspective of the woman submitting to an ultrasound examination. This helped me to understand the central tension at play in the clinical encounter described at the opening of this essay, a tension that arises from the medical need to be an objective, detached witness, and the woman’s need to be seen as a whole person.

The depictions of maternal imagining following ultrasound examination in the texts I have studied, together with my own experiences as an expectant mother, make me feel certain that the emotional responses begin pre-birth, powerfully mediated by what is seen on the screen. My work, as an expert witness engaged in scrutinizing maternal-child relationships, meant that attachment theory, used daily in my clinical practice, inevitably became a lens that I used to look at these texts. Attachment theory was developed in the 1960s by Mary Ainsworth, a North American childhood-developmental psychologist who was the first person to investigate the attachment bonds between babies and their mothers. Together with her colleague John Bowlby, a British psychiatrist and psychoanalyst, Ainsworth developed a theory of infant-maternal attachment, postulating that newborn babies seek out the care of one main parenting figure, usually the mother, and that a mother’s emotional responses to her baby influence many aspects of the baby’s development.

These thoughts about pre-birth attachment have been influenced by pediatrician and psychoanalyst Donald Winnicott, who postulated that emotional bonds between mothers and children are formed by “an ordinary mother who is fond of her baby” (Winnicott 1952, 39). In my clinical work, I observed mothers gazing at babies and attended to whether their babies responded positively, by snuggling or smiling. This gaze-and-response creates a reciprocal positive feedback loop—the “maternal-infant dance”—from which attachment develops. Helping parents to develop positive images of their infant increases the probability of a secure parent–child attachment relationship.

As a psychiatrist, I was taught to observe mothers locking eyes with infants, but I have also come to appreciate the emotional significance of women gazing at their ultrasound prints. Winnicott developed attachment theory before the advent of routine pregnancy ultrasounds. However, these three texts have left me newly alert to the idea that seeing a fetus on a screen in real time directs an expectant mother’s imagination about that child.

In her book *Visual Methodologies: An Introduction to Researching with Visual Materials* (2012), sociologist Gillian Rose asserts “looking, seeing and knowing have become perilously intertwined” (Rose 2012, 5). All three protagonists’ descriptions present a similar emotional dance between a mother and her unborn child.

As a reader, I had a strong sense of the different meanings of fetal images in obstetric and maternal contexts, especially through the point of view of the women viewing their images. Rose described this as “audiencing,” defined as “the process by which a visual image has its meanings renegotiated by particular audiences in specific circumstances” (Rose, Gillian. 2012, 30). Women lying on examining couches are akin to a theater audience; their role is clear from the moment the clinical curtain is drawn back. As the controversy with which I began this paper makes plain: from the obstetric perspective, pregnant women are expected to audience, not to act.

With Rose’s definitions in mind, these three texts can be seen as refocusing perspectives, from clinical vision onto maternal visuality. While the ultrasound gives vital clinical information to the clinicians in the texts, it scaffolds each protagonist’s fantasies of mothering. Reading these texts, I came to appreciate that a degree of clinical detachment is inherent in any act of obstetric scrutiny. I also reconsidered that my own sense of maternal attachment was bolstered by gazing at my unborn child on ultrasound.

What women see on ultrasound can change our lives in the blink of an eye. The heartbeat we do not hear in ultrasound rooms can shatter our lives. In each text, the woman’s maternal imagining is described in the immediate aftermath of the clinical image, each woman merging observations from the ultrasound with mental images of children who are not born. The protagonists do not imagine an unviable fetus, but their specific unborn. In each woman’s imagination, the maternal–fetal relationship is plain. In the obstetric scan image, the woman’s presence is murky, her maternal status obfuscated, and the maternal relationship obliterated. Our experiences in the ultrasound suite also change how we are seen and perceived by others, molding us like unfired clay. In these texts, it is during, or immediately after, the experience of ultrasound that the scanned women form a mental image of their unborn child, including, in Mantel’s memoir, a ghost child. Perhaps these mental images existed before in women’s minds, but they are certainly solidified during the experience of ultrasound. While the ultrasound gives vital clinical information to all three clinicians in the texts, it scaffolds each protagonist’s fantasies of mothering, giving them strength and durability.

Women’s writing reclaims a much-needed sense of humanity from the obstetric discourse. These three literary texts were not written to present counterpoints to scientific papers, yet, nevertheless, I hope that my analysis encourages clinicians to consider the relational aspects of miscarriage, perhaps reframing a medical event as a relational loss, a maternal grief. In the three literary texts I have presented in this paper, women audiencing their ultrasound scans after miscarriage conceive of themselves as bereft of the opportunity of motherhood. Even as ultrasound enables clinicians to image the unborn, it shapes maternal desires and mediates experiences of the maternal–fetal relationship, including maternal grief. As Mantel asks, “What’s to be done with the lost, the dead, but write them into being?” (Mantel 2013, 231).

## Data Availability

N/A.

## References

[CR1] Carty-Williams, Candice. 2019. *Queenie*. London: Trapeze.

[CR2] Mantel, Hilary. 2013. *Giving up the ghost: A memoir*. London: Fourth Estate.

[CR3] O’Farrell, Maggie. 2019. *I am, I am, I am: Seventeen brushes with death.* London: Vintage.

[CR4] Rose, Gillian. 2012. *Visual methodologies: An introduction to researching with visual materials*. 3rd ed. London: SAGE.

[CR5] Winnicott, Donald. 1952. Letter to Roger Money-Kyrle, 27th November. In *The spontaneous gesture: Selected letters of D. W. Winnicott,* 38-43*.* London: Karnac Books.

